# Modification of Urea-Formaldehyde Resin with Triethylenetetramine: Effect on Adhesive Properties and Plywood Strength

**DOI:** 10.3390/polym17192652

**Published:** 2025-09-30

**Authors:** Jakub Kawalerczyk, Dorota Dukarska, Błażej Góral, Petar Antov, Dorota Dziurka, Radosław Mirski

**Affiliations:** 1 Department of Mechanical Wood Technology, Faculty of Forestry and Wood Technology, Poznań University of Life Sciences, 60-627 Poznań, Poland; 2 Faculty of Forest Industry, University of Forestry, 1797 Sofia, Bulgaria; p.antov@ltu.bg

**Keywords:** plywood, urea-formaldehyde resin, formaldehyde scavenger, triethylenetetramine

## Abstract

Due to its multiple amino groups, triethylenetetramine (TETA) can be used as an effective formaldehyde scavenger contributing to the reduction in formaldehyde emission from plywood. This study aimed to evaluate the effect of small TETA loadings on the properties of urea-formaldehyde (UF) resin and the performance of the resulting plywood. Adhesive mixtures containing 0%, 0.5%, 1.0%, and 1.5% TETA were prepared and characterized in terms of pH, viscosity, solids content, and gel time. The incorporation of TETA significantly increased adhesive pH and gel time, while viscosity and solid content were not significantly affected. The analysis of formaldehyde content and spectroscopic and thermogravimetric analyses of the cured adhesives showed reduced formaldehyde content, changes in chemical structure, and enhanced thermal stability at lower temperatures but accelerated degradation at higher temperatures. Formaldehyde emission from plywood was reduced; however, bonding quality and mechanical performance decreased with higher TETA content. Nevertheless, the wet shear strength of all variants exceeded 1 N/mm^2^. Adhesive formulation containing 0.5% TETA was selected as the optimal variant, providing environmental benefits while maintaining satisfactory plywood performance.

## 1. Introduction

In the second decade of the 21st century, the global production of wood-based panels reached 368 million m^3^, of which plywood accounted for 118.4 million m^3^, representing approx. 30% of the total production [1]. Due to its layered structure, plywood has several advantages over solid wood, including high bending and tensile strength, lower susceptibility to stresses and moisture-related deformations, and good stiffness [2,3]. The properties of plywood are strongly influenced by the wood species used, the type of resin applied, and the technological process of veneer production [4,5,6]. Because of these favorable characteristics, plywood has a wide range of applications, including in the furniture industry, construction (as both structural and finishing material), packaging production, boatbuilding, and land transport manufacturing. The selection of a specific resin for plywood production depends primarily on the intended application of the plywood and the environmental conditions in which it will be used [7].

Urea-formaldehyde (UF) resin used in the production of interior-grade plywood is synthesized from two main components: urea (CH_4_N_2_O) and formaldehyde (HCHO). It is the most widely applied adhesive for plywood intended for indoor applications [8], mainly because of its high availability, low cost, short pressing time, and the light color of the glue line. However, UF resins also exhibit significant drawbacks, including low water resistance and formaldehyde emission [9]. Formaldehyde is a colorless gas with a strong, irritating odor, highly reactive even at room temperature [10]. It plays a crucial role in the curing reaction of UF resin, enabling the formation of a cross-linked adhesive structure during plywood pressing [11,12]. However, formaldehyde is released both during hot pressing and throughout the service life of manufactured wood-based panels. Its emissions are harmful to human and animal health, potentially causing immune system disorders, respiratory diseases, and, under long-term exposure, even cancer. Other reported effects of formaldehyde exposure include reduced concentration, eye irritation, and drowsiness [13,14,15]. Because of these adverse impacts, numerous efforts by both industry and the scientific representatives worldwide are aimed at reducing formaldehyde emissions from widely used wood-based materials.

In recent years, there has been a growing number of studies in which UF resin is modified with various chemical compounds to reduce the amount of free formaldehyde released from plywood and other wood-based materials [16]. The modifiers can be either organic or inorganic. Furthermore, they may take the form of chemical substances, such as amine compounds that bind formaldehyde, or various types of fillers, such as plant tannins, flours, or activated carbon, which are added to regulate the viscosity of the adhesive [16,17]. However, according to Hassannejad et al. [18], chemical compounds containing the amine group are the most effective for formaldehyde adsorption.

The addition of various amines has been the subject of several studies on wood-based materials. Ghani et al. [19,20], studied the effect of adding methylamine, ethylamine, and propylamine to UF resin on particleboard properties. Amines improved thermal stability and significantly reduced formaldehyde emissions, with propylamine and ethylamine achieving E0-class boards. However, amine addition increased water absorption and thickness swelling, while reducing bending strength, modulus of elasticity, and internal bond. Propylamine has also been investigated as a UF resin modifier in plywood production, and its addition was found to reduce formaldehyde emissions. However, higher amounts caused significant decrease in bonding quality, bending strength, and modulus of elasticity, indicating that the modifier should not exceed 1% [21]. Boran et al. [22] investigated the mechanical and physical properties of MDF panels bonded with UF resin modified with various amines, including propylamine, methylamine, ethylamine, and cyclopentylamine. The addition of propylamine, methylamine, and cyclopentylamine improved the physical and mechanical properties of the boards, whereas ethylamine and urea negatively affected performance. Proper use of modifiers such as propylamine and methylamine showed considerable potential for reducing formaldehyde emissions from wood-based materials. Gao et al. [23] investigated the use of ammonium pentaborate (APB) to modify UF resin for plywood production. Addition of 2–8% APB significantly extended the resin gel time but markedly reduced formaldehyde content in the cured resin and emissions from the resulting plywood, with reductions up to approx. 79–80%. However, APB adversely affected bonding quality, especially for resins with low formaldehyde-to-urea ratios. Valyova et al. [24] showed that using 2% ammonium sulfate as a hardener for UF resin, combined with the addition of urea or ammonium bicarbonate (1–3%) as formaldehyde scavengers, effectively reduced formaldehyde emission from plywood, with urea lowering it by approx. 54% and ammonium bicarbonate by approx. 22%.

Previous studies on UF resin modification have focused primarily on monofunctional amines such as methylamine, ethylamine, or propylamine, while the effect of triethylenetetramine (TETA) has not yet been investigated. Due to its multiple amino groups, TETA can act as an effective formaldehyde scavenger by reducing free formaldehyde emission from cured resin. Therefore, this study aimed to evaluate the influence of small additions of TETA on the properties of UF resin and on the performance of the resulting plywood.

## 2. Materials and Methods

### 2.1. Materials

The urea-formaldehyde (UF) adhesive was supplied by a local industrial plywood manufacturer and was characterized by a solid content of 61.3%, a gel time of 241 s at 100 °C, a viscosity of 853 mPa·s, a pH of 7.6. Triethylenetetramine (analytical grade) was obtained from Merck (Poznań, Poland). To adjust the adhesive viscosity, commercially available rye flour was added as an extender (Melvit, Warsaw, Poland). Ammonium nitrate (analytical grade), introduced as a hardener, was purchased from Chempur (Piekary Śląskie, Poland). Rotary-cut birch (*Betula* L.) defect-free veneers, with an average density of approx. 600 kg/m^3^, dimensions of 320 mm × 320 mm × 1.4 mm, a moisture content of 5 ± 1%, were used for plywood manufacturing.

### 2.2. Adhesive Mixtures Preparation and Testing

The adhesive mixtures contained varying amounts of TETA (0%, 0.5%, 1.0%, and 1.5%) and were designated as REF, T0.5, T1.0, and T1.5, respectively. Additionally, 3% of a 20% aqueous ammonium nitrate solution was added as a hardener to all variants. Moreover, to adjust the viscosity of the mixtures, 10% rye flour was also added as an extender. After the addition of all components, the mixtures were manually stirred until a homogeneous mixture was achieved.

Viscosity was determined using a Brookfield DV-II+Pro rotary viscometer (Middleboro, MA, USA). The pH was measured with a Testo 206 pH meter (Pruszków, Poland). Solids content and gel time at 100 °C were assessed following the standards EN 827 [25] and PN-C-89352-3 [26], respectively. Each parameter was determined in triplicate.

The formaldehyde content in the cured adhesive was determined in triplicate using the perforator method according to EN 120 [27], adjusted following the procedure of Dziurka and Mirski [28]. A sample of 5 ± 0.01 g was subjected to extraction with boiling toluene in a perforator apparatus. The formaldehyde concentration in the resulting solution was quantified spectrophotometrically using the ammonium acetate and acetylacetone method, with absorbance measured at 412 nm on a Biosens UV-5600 spectrophotometer (Warsaw, Poland).

The chemical structure of cured reference and TETA-modified variants was assessed with the attenuated total reflectance—Fourier transform infrared spectroscopy (ATR-FTIR) using Spectrum Two FTIR spectrometer equipped using a Universal ATR with a diamond crystal (PerkinElmer, Waltham, MA, USA).

The thermal stability of the cured adhesive mixtures was assessed by thermogravimetric analysis (TGA) using a TGA4000 thermogravimeter (PerkinElmer, Waltham, MA, USA). Samples were heated to 600 °C at a rate of 10 °C/min under a nitrogen atmosphere with a flow rate of 20 mL/min. The analyses were performed on the reference formulation and on formulations with the lowest and highest modifier contents (0.5% and 1.5%).

### 2.3. Plywood Manufacturing and Testing

Adhesive mixtures were applied to the surface of the external veneers at a spread rate of 160 g/m^2^ to produce three-layer plywood. The assembled sets were hot-pressed for 4 min at 120 °C under a pressure of 1.6 MPa. Four plywood panels were manufactured for each variant.

To evaluate adhesive performance in plywood, bonding quality was determined by shear strength testing in both dry state and after 24 h of soaking in water at 20 ± 3 °C according to EN 314-1 [29], using 12 samples from each variant. Mechanical properties of the resulting materials were evaluated based on three-point bending strength (MOR) and modulus of elasticity (MOE) investigated in both parallel and perpendicular directions to the grains of the outer veneer according to EN 310 [30], using 12 samples from each variant. Formaldehyde emission was determined in triplicate using the flask method according to EN 717-3 [31]. Formaldehyde content in the collected solution was measured at 412 nm using the ammonium acetate and acetylacetone method on a Biosens UV-5600 spectrophotometer (Warsaw, Poland).

### 2.4. Statistical Analysis

To analyze the obtained results, a one-factor ANOVA was performed using Statistica 13.0 software. For adhesive mixtures parameters, the Kruskal–Wallis test (α = 0.05) was applied to assess the statistical significance of differences, and the Tukey post hoc test (α = 0.05) was used for evaluating differences in plywood properties.

## 3. Results and Discussion

**Figure 1:**
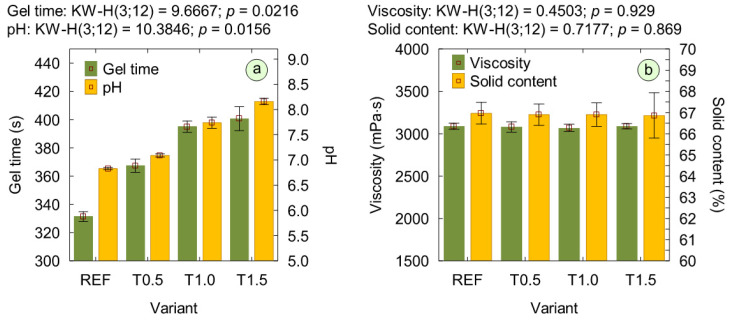
Properties of adhesive mixtures: (**a**) gel time and pH; (**b**) viscosity and solids content.

Figure 1a shows the results of pH measurements of the adhesive mixtures. The outcomes indicate that the addition of the modifier led to an increase in the pH of the adhesive mixtures. The pH values rose with increasing TETA content, which can be attributed to the strongly basic nature of the modifier. As a consequence of the pH increase, the modified variants exhibited a prolonged gel time (Figure 1a). It was demonstrated that the addition of only 0.5% of TETA caused a significant prolongation of the gel time by more than 30 s compared to the reference variant. Moreover, for the T1.0 and T1.5 variants, the gel time remained at a similar level, approx. 60 s longer than observed for the reference mixture. This is consistent with the findings of Ghani et al. [20], who also observed a prolongation of the gel time of UF resin modified with amines in particleboard production. They reported that the addition of modifiers such as methylamine, ethylamine, or propylamine increased the gel time from 65 s to 240–306 s, depending on the type and amount of the introduced TETA. Furthermore, the viscosity and solids content of the adhesive mixtures are presented in Figure 1b. The viscosity of the adhesive mixture is a crucial parameter in plywood production technology, directly affecting its properties [32]. It was found that all mixtures exhibited viscosities suitable for plywood production, which, according to Zhang et al. [33] and Soubam et al. [34], can reach up to 25,000 mPa·s. Moreover, based on statistical analysis, no significant effect of the performed modification on the viscosity and solids content of the mixtures was observed. Most likely, the modifier loading range between 0.5 and 1.5% was insufficient to cause significant changes in these parameters of the adhesive.

**Figure 2:**
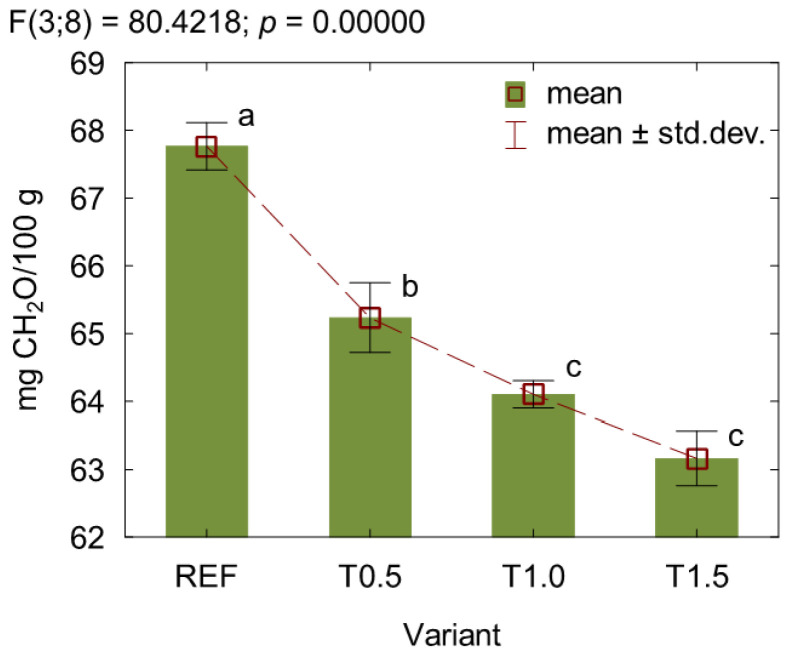
Formaldehyde content in the cured adhesive mixtures. (a, b, c letters mark homogenous groups in the HSD Tukey test).

The results of formaldehyde content analyses in the cured adhesive mixtures are presented in Figure 2. It was found that the addition of TETA significantly reduced the formaldehyde content compared to the reference variant. Moreover, this effect became more pronounced with increasing loading of the modifier. Compared to the REF variant, the formaldehyde content decreased by approx. 4% for variant T0.5, approx. 6% for variant T1.0, and approx. 7% for variant T1.5. The observed formaldehyde-scavenging effect was attributed to the presence of amino groups in TETA, which, according to Hassannejad et al. [18] can react with both free formaldehyde in the resin and hydrolyzed formaldehyde in the resulting material. When amine reacts with an aldehyde, the reaction typically follows an addition-elimination mechanism. Initially, the amine nitrogen attacks the carbonyl carbon, forming a hemiaminal through a proton transfer from nitrogen to oxygen. Next, the hemiaminal undergoes protonation and loses a water molecule, and a final deprotonation step produces imine [20,35,36].

**Figure 3:**
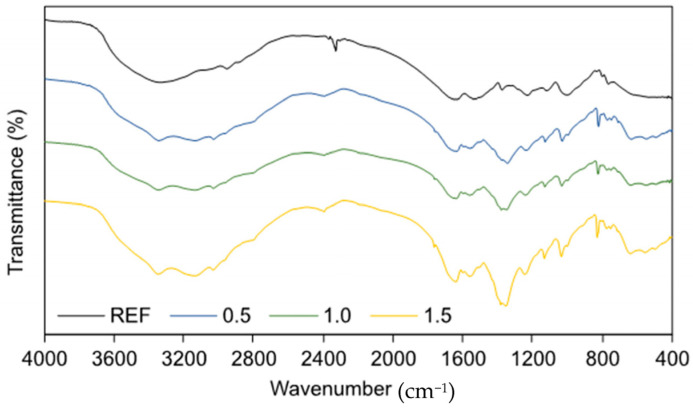
ATR-FTIR spectra of cured adhesive mixtures.

The FTIR spectra of both reference and TETA-modified UF resin variants are presented in Figure 3. The reference resin exhibited characteristic UF peaks, including a broad band at 3355 cm^−1^ corresponding to O–H and N–H stretching vibrations, symmetric and asymmetric C–H stretching vibrations at 2953 and 2882 cm^−1^, respectively, and a band at 1645 cm^−1^ attributed to C=O stretching [37,38,39,40]. Modification with TETA led to an increase in the intensity of the band at 3335 cm^−1^, likely due to overlapping vibrations from the OH and NH_2_ groups of the modifier [41], while the peak at 2960 cm^−1^, corresponding to asymmetric –CH stretching, decreased, possibly reflecting extensive conversion of –CH_2_OH groups. A similar effect was observed as a result of the addition of ammonium-based hardeners [42]. A small peak at 1443 cm^−1^, attributed to C–H bending vibrations [43], appeared in the modified variants, which may be related to structural changes in the polymer induced by TETA incorporation. The spectra also showed a weak peak at 1610 cm^−1^, indicative of C=N stretching, suggesting formation of imine bonds during the reaction between amino and aldehyde groups [44]. A peak at 995 cm^−1^, associated with methylol groups [45], indicates the formation of new linkages resulting from reactions between TETA amino groups and formaldehyde. The increased intensity of the band at 830 cm^−1^, corresponding to C–O–C stretching [46], reflects additional network linkages resulting from interactions between hydroxymethyl groups and amino groups of the modifier, while a small peak at 768 cm^−1^, attributed to NH_2_ wagging [47], confirms the incorporation of TETA amino groups into the UF resin matrix. Overall, the FTIR analysis indicates chemical changes characteristic of amine modification of UF resin.

**Figure 4:**
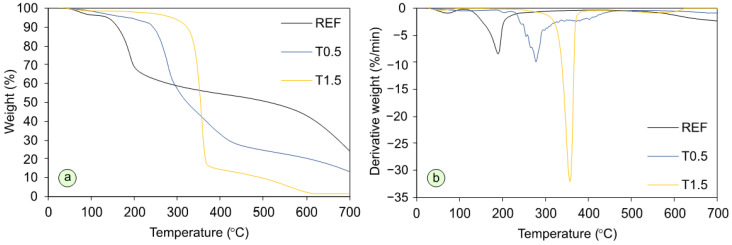
TG (**a**) and DTG (**b**) curves of cured adhesive mixtures.

As shown in Figure 4 and Table 1, the applied modification significantly affected thermal stability of cured adhesives. In the first stage, corresponding to a weight loss up to approx. 220 °C, which results from the removal of water and free formaldehyde from the resin, the thermal degradation proceeded considerably slower for the modified variants. Moreover, in this range thermal stability increased with higher TETA content. This effect is consistent with the observations of Ghani et al. [20], who also reported increased thermal stability of UF resin after modification with propylamine, ethylamine, and methylamine. The authors attributed this effect to the reaction of amines with free formaldehyde in the resin. However, in the case of TETA modification, this effect was observed only at lower temperatures. Interestingly, at temperatures above 300 °C, where according to Qi et al. [48], the greatest mass loss occurs due to the breakdown of methylene and methylene ether bonds, the modified variants experienced a rapid and pronounced weight loss. The intensity of the observed changes increased with higher modifier content. This may indicate disruptions in the resin structure, considering that the reduced reactivity, evidenced by a significant prolongation of the gel time, can lead to a lower degree of conversion and reduced cross-linking density in the cured UF resin [49]. Moreover, according to Lee and Kim [50], the addition of scavengers significantly reduces the crystallinity of UF resin, which may also contribute to a decrease in its thermal stability. At temperatures above 400 °C, slow further decomposition and pyrolysis of the resin and its residues progressed [48].

**Figure 5:**
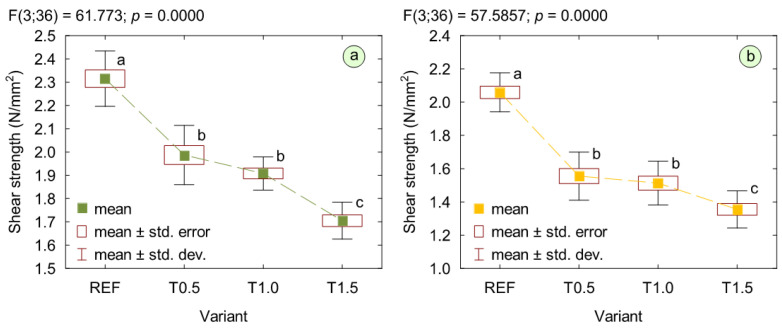
Bonding quality of plywood: (**a**) dry shear strength; (**b**) wet shear strength. (a, b, c letters mark homogenous groups in the HSD Tukey test).

The results of the determinations of bonding quality in plywood, conducted both in dry conditions and after water soaking, are presented in Figure 5. The outcomes indicate that the addition of TETA to the adhesive mixture reduced the strength of the adhesive bond lines. Compared with the reference plywood, the dry shear strength values for variants T0.5, T1.0, and T1.5 decreased by 13%, 17%, and 25%, respectively. Furthermore, after water soaking, the shear strength decreased by 24% in variant T0.5, by 26% in T1.0, and by 33% in T1.5 compared with the reference. Statistical analysis revealed no significant differences between the variants containing 0.5% and 1% of the modifier; however, increasing its content to 1.5% resulted in a further statistically significant reduction in bonding quality. The observed deterioration was most likely caused by the reduced reactivity of the adhesive mixture resulting from a considerable increase in its pH. According to Xing et al. [51], a pH value of around 7 is a critical point, and exceeding this value may significantly slow down the polycondensation reaction, resulting in a not fully crosslinked structure characterized by reduced strength. Moreover, considering that the hydrolytic degradation of the resin depends on its cross-linking degree [52], the applied modification may also have reduced the water resistance of the bond lines. It is also important to note that the incorporation of a scavenger with multiple amino groups may lead to premature reaction with free formaldehyde, reducing the amount of formaldehyde available for crosslinking within the UF network [53]. Nevertheless, the wet shear strength of all plywood variants exceeded 1 N/mm^2^.

**Figure 6:**
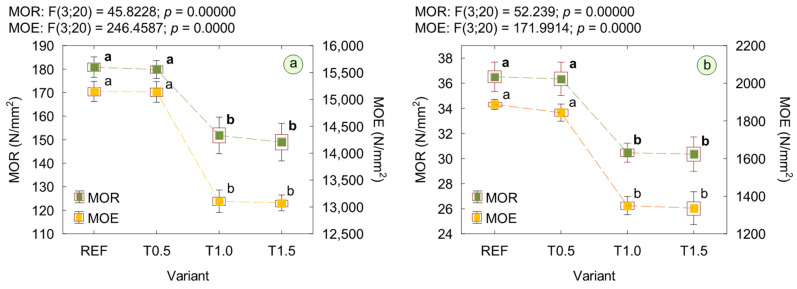
Mechanical properties of plywood: (**a**) parallel to the grain; (**b**) perpendicular to the grain. (a, b letters mark homogenous groups in the HSD Tukey test).

The results of the mechanical properties of plywood, including MOR and MOE tested parallel and perpendicular to the grain of the outer veneer, are presented in Figure 6. The results showed that the addition of 0.5% TETA to the UF resin did not cause statistically significant changes in strength, neither parallel nor perpendicular to the grain. However, for variants T1.0 and T1.5, a significant decrease in MOR was observed, with reductions of approx. 16% for both variants and in both directions. Similarly, the addition of 0.5% TETA did not lead to significant changes in MOE of plywood, regardless of the anatomical direction of the outer veneer. For variants T1.0 and T1.5, however, MOE decreased by approx. 13% parallel to the grain and by approx. 28% perpendicular to the grain, compared with the control plywood. No statistically significant changes in MOR or MOE were observed between the variants containing 1% and 1.5% TETA in adhesive formulations. Considering that one of the factors affecting the flexural characteristics of plywood is the strength of the adhesive bond lines [54], the observed reduction most likely was caused by a decrease in the bonding quality of the resulting material.

**Figure 7:**
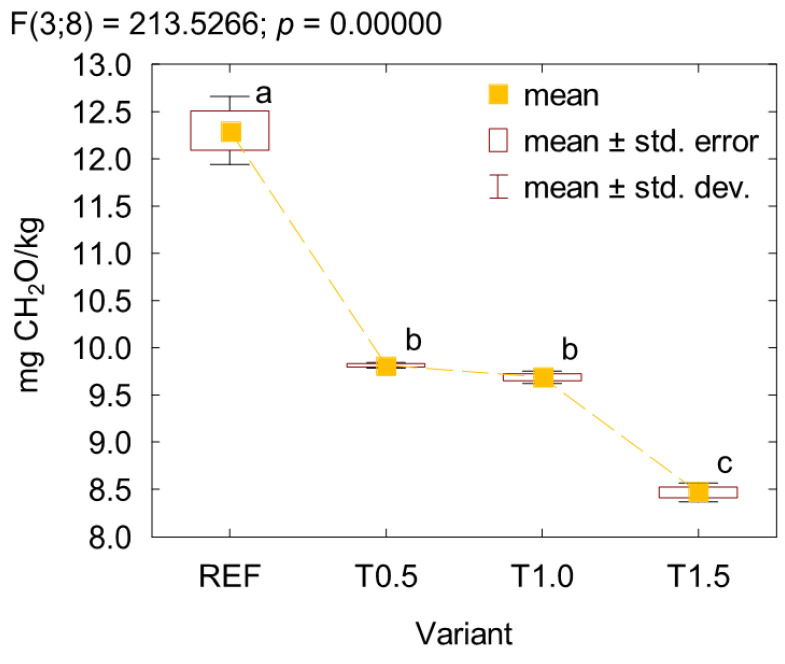
Formaldehyde emission of plywood. (a, b, c letters mark homogenous groups in the HSD Tukey test).

Figure 7 shows the formaldehyde emission results of the plywood. The outcomes revealed that the addition of 0.5% and 1% TETA reduced formaldehyde emission by approx. 20%. A further increase in the modifier content to 1.5% led to an additional significant reduction of about 30% compared with the reference plywood. The observed changes resulted from a decrease in free formaldehyde content in the UF resin due to its reaction with the amine groups of TETA. The observed changes are very similar to the effect of adding propylamine, where a 1% addition reduced plywood formaldehyde emission by approx. 20%, and 1.5% by about 30%. Therefore, considering the limited amount of free formaldehyde remaining in the UF resin, the number of amino groups in the modifier is not always crucial or necessarily predictive in terms of a stronger formaldehyde-scavenging effect, especially taking into account structural differences affecting the accessibility of the amino groups.

## 4. Conclusions

The incorporation of TETA into UF resin significantly influenced its properties and the performance of plywood. The modification increased the pH of the adhesive, leading to prolonged gel time, while viscosity and solid content remained unaffected and within the range suitable for plywood production. The presence of amino groups in TETA effectively reduced the free formaldehyde content in the cured adhesive mixture and resulted in lower formaldehyde emissions from plywood. ATR-FTIR analysis showed that the TETA modification caused chemical changes related to the incorporation of amine groups and their reactions with formaldehyde. Thermogravimetric analysis of cured adhesives showed that the modification enhanced stability at lower temperatures, but at higher temperatures, the modified variants degraded more rapidly, most likely due to a disrupted adhesive structure. In plywood, the incorporation of TETA decreased bonding quality and mechanical performance, particularly at higher loadings, which can be attributed to slower polycondensation and the hindered formation crosslinking network. Nevertheless, for all tested variants, the wet shear strength of the plywood exceeded 1 N/mm^2^. In conclusion, TETA acts as an efficient formaldehyde scavenger in UF resins, but its use leads to deterioration of plywood strength. Therefore, careful optimization of the modifier content is essential to achieve a balance between environmental benefits and satisfactory performance of the final product. The 0.5% TETA addition was considered the optimal variant, taking into account the modification costs and the unaffected mechanical properties (MOR and MOE), and formaldehyde emission reduced to a similar level as the 1% addition.

## Figures and Tables

**Table 1:** Thermal decomposition parameters.

Variant	Weight Loss (%)									Resiude at 700 °C (%)
	100 °C	150 °C	200 °C	250 °C	300 °C	350 °C	400 °C	500 °C	600 °C	
REF	3.55	7.03	31.16	37.90	41.23	43.55	45.43	49.28	56.98	24.28
T0.5	1.55	3.72	1.06	11.94	42.94	56.25	66.86	75.43	79.69	13.27
T1.5	1.39	1.49	2.02	2.98	6.19	39.33	85.58	90.3	97.70	1.51

## Data Availability

The raw data supporting the conclusions of this article will be made available by the authors on request.
